# Survey Results on Pathologic Aspects of Endocervical Adenocarcinoma by the International Society of Gynecological Pathologists

**DOI:** 10.1097/PGP.0000000000000744

**Published:** 2021-02-09

**Authors:** W. Glenn McCluggage, Joseph T. Rabban, Naveena Singh, Esther Oliva

**Affiliations:** Department of Pathology, Belfast Health and Social Care Trust, Belfast (W.G.M.); Department of Cellular Pathology, Barts Health NHS Trust, London (N.S.), UK; Pathology Department, University of California San Francisco, San Francisco, California (J.T.R.); Pathology Service, Massachusetts General Hospital, Harvard Medical School, Boston, Massachusetts (E.O.)

**Keywords:** Cervical adenocarcinoma, Grossing, Processing, Diagnosis, Reporting, Survey

## Abstract

The International Society of Gynecological Pathologists (ISGyP) undertook a project to provide evidence-based recommendations for pathologic reporting of all aspects of endocervical adenocarcinoma. The first step in the process was the design of an extensive survey to collect baseline information on existing practices regarding grossing, processing, diagnosing, and reporting of endocervical adenocarcinoma among the members of the society. The web-based survey of 98 questions was emailed to all members of ISGyP and there were 175 respondents (38.5% of ISGyP members). The responses, as expected, revealed areas of uniformity but also areas of substantial variation. The results of the survey are presented herein and assisted in developing the recommendations presented in the other reviews in this issue.

Cervical carcinoma is the fourth most common malignancy in women worldwide 1. In 2018, there were an estimated 569,847 new cases diagnosed, representing 6.9% of all cancers in women 1. In the developed world, cervical cancer is less common than endometrial and tubo-ovarian cancer but is more common than these neoplasms in under-resourced countries 1. While ~75% to 80% are squamous cell carcinomas, nearly all of which are associated with oncogenic high-risk human papillomavirus (HPV-associated), adenocarcinomas are increasing in incidence both in real and apparent terms 2–4. Part of this is secondary to the decrease in incidence of cervical squamous cell carcinoma in developed countries due to organized cervical screening programs which have not had the same impact on adenocarcinomas.

The International Society of Gynecological Pathologists (ISGyP) undertook a major project on all aspects of the pathologic reporting of cervical adenocarcinomas, similar to that recently undertaken for endometrial carcinomas 5,6. Part of the impetus for the project was related to 3 significant recent developments regarding pathologic aspects of these neoplasms, including a new international classification of cervical adenocarcinomas predominantly based on the presence or absence of morphologic features associated with oncogenic HPV, the recommendation for a pattern-based classification of invasion in HPV-associated adenocarcinomas, and an updated FIGO staging system for cervical carcinomas 7.

There have been no large-scale studies documenting current practices for grossing, processing, diagnosis, reporting, and ancillary testing of cervical adenocarcinomas among gynecologic pathologists. As an initial step in the cervical adenocarcinoma project, the ISGyP undertook a survey of its members to investigate current practice and gather baseline information; the results are presented herein.

## MATERIALS AND METHODS

A 98-question survey was designed by 4 members of the organizing committee of the project (the authors of this manuscript). The survey was modified and approved by all members of the organizing committee. The questions covered the demography of the respondents and information regarding current practice and opinions relating to specimen grossing and processing, intraoperative assessment, reporting of various parameters, and ancillary testing of cervical adenocarcinomas. The questions varied in format, including some with binary responses (eg, yes/no), some single choice responses from a list of possibilities and others multiple possible selections from a list of possibilities. For some questions, respondents were asked to expand on their responses in the form of comments. All respondents were required to answer all the questions.

A link to the survey was emailed to the ISGyP membership (n=457). Participants were given a 6-wk deadline to complete the survey, with a reminder sent over that time-period. An incentive to undertake the survey was that participants would be invited to an ISGyP workshop regarding the project that coincided with the 2020 annual United States and Canadian Academy of Pathology meeting in Los Angeles (February 2020).

## RESULTS

There were 175 respondents to the survey representing 38.5% of the total society membership. Respondents came from 32 countries with 51.1% from North America, 26.1% from Europe, and the remainder from other countries; these percentages are broadly reflective of the international membership of ISGyP. Many, but not all, of the questions and responses are provided in Tables 1–9.

**TABLE 1:** Demographics of respondents to survey and general questions

In which continent do you currently practice?	North America (51.1%): Europe (26.1%); Asia (8.5%); Australasia (7.5%); Africa (3.4%); South America (3.4%)
What is your practice setting?	Academic institution (77.3%); community/private practice (17%); currently a trainee (1.8%); other (3.9%)
How many years have you been in practice?	<5 (11.9%); 5–10 (23.3%); 11–15 (10.8%); 16–20 (13.1%); 21–25 (11.9%); 26–30 (10.2%); >30 (15.9%)
Are you a specialist or generalist?	Gynecologic pathology only (26.7%); gynecologic pathology and 1 or 2 other specialties (60.2%); general surgical pathology (9.7%); other (3.4%)
What percentage of your workload comprises gynecologic oncology cases?	<5% (0.8%), 5%–10% (4.9%), 11%–25% (18.2%), 26%–50% (29.5%), 51%–75% (25%), 76%–100% (21.6%)
What type of specimens do you see in your practice?	Biopsies and resections (97.7%); biopsies only (1.7%); resections only (0.6%)
Are you familiar with the ICCR reporting recommendations for reporting cervical cancer?	Yes (80.7%); no (19.3%)
Do you use the ICCR recommendations for reporting cervical cancer?	Yes (51.7%); no (48.3%)
Are you aware of the CAP cancer protocol for reporting cervical cancer?	Yes (88.6%); no (11.4%)
Do you use the CAP recommendations for reporting cervical cancer?	Yes (70.5%); no (29.5%)

CAP indicates College of American Pathologists; ICCR, International Collaboration on Cancer Reporting.

**TABLE 2:** Questions and responses regarding tumor grossing and processing

Who grosses cervical loop/cone biopsies in your institution?	You (18.8%); pathology assistant/laboratory technician (66.5%); trainees (76.7%)
Which method do you use for grossing loop/cone biopsies?	Serial radial embedding (clock-face) (70.5%); serial sectioning in sagittal/parasagittal planes (26.1%): other (3.4%)
Who grosses hysterectomy specimens for cervical cancer in your institution?	You (24.4%); pathology assistant/laboratory technician (56.8%); trainees (79.5%)
Do you ink cervical loop/cone biopsies?	Fragmented unorientated specimens (51.1%); intact cone specimens (92%); no (6.8%)
Do you ink parametrial resection margins in hysterectomy/trachelectomy specimens for cervical cancer?	Yes (90.9%); no (9.1%)
Do you ink vaginal resection margins in hysterectomy/trachelectomy specimens for cervical cancer?	Yes (88.1%); no (11.9%)
Do you ink the upper endocervical resection margin in trachelectomy specimens for cervical cancer?	Yes (88.6%); no (11.4%)
Do you harvest tissue for research, molecular studies, biobanking, etc?	Yes, routinely (15.9%); yes, sometimes (51.7%); no (32.4%)
Are hysterectomy/trachelectomy specimens for cervical cancer fixed before grossing?	Yes (90.3%); no (9.7%)
For cervical loop/cone biopsies, do you record the dimensions of all pieces of tissue in the pathology report?	Yes (97.7%); no (2.3%)
For cervical loop/cone biopsies, do you record whether the tissue is an intact loop or a strip?	Yes (93.8%); no (6.2%)
For hysterectomy specimens, do you report the length of the vaginal cuff?	Yes (maximum and minimum lengths) (48.3%); yes (single dimension) (44.3%); no (7.4%)
For hysterectomy specimens, do you report a measurement of the parametrial tissue?	Yes (horizontal and vertical dimensions) (52.8%); yes (horizontal dimensions only) (13.6%); yes (vertical dimensions only) (2.3%): no (31.3%)
For hysterectomy specimens do you record the site of the tumor?	Always if grossly visible (59.1%): always (39.2%); no (1.7%)
For hysterectomy/trachelectomy specimens, do you submit the entire parametrial tissue for histologic examination?	Yes (77.8%); no (22.2%)
For hysterectomy/trachelectomy specimens, do you submit the parametrial tissue separately from the cervix?	Yes (88.6%); no (11.4%)
For hysterectomy/trachelectomy specimens, how much of the tumor do you submit?	The entire tumor (26.1%); representative sections (1 block per cm of tumor) (42%); representative sections (other) (31.8%)
For hysterectomy/trachelectomy specimens, if the tumor is not grossly visible, do you submit the entire cervix?	Yes (97.7%); no (2.3%)
For hysterectomy specimens for cervical cancer, how much of the lower uterine segment do you submit if grossly normal?	Entire lower uterine segment (13.6%); 1 section of anterior and 1 of posterior half (75%); other (11.4%)
For hysterectomy specimens for cervical cancer, how many sections of grossly normal uterine corpus do you submit?	Entire endometrium (0%); 1 section of anterior and 1 of posterior half (80.7%); other (19.3%)
For hysterectomy specimens for cervical cancer, how many sections of grossly normal ovary do you submit?	Entire ovary (11.9%); one half of the ovary (25%); 1 section (36.9%); 2 sections (21%); more than 2 blocks (5.2%)
For hysterectomy specimens for cervical cancer, how many sections of grossly normal fallopian tube do you submit?	1 (15.9%): 2 (21%); >2 (17.6%); all tube (9.1%); all tube using SEE-FIM protocol (36.4%)
For hysterectomy specimens for cervical cancer, do you use large tissue (macro) blocks?	Yes (10.8%); no (89.2%)

**TABLE 3:** Questions and responses regarding intraoperative tumor assessment

How often do you perform gross intraoperative examination (without frozen section) for cervical adenocarcinoma?	Always for hysterectomy specimens (4.5%); sometimes for hysterectomy specimens (25%); always for trachelectomy specimens (5.7%); sometimes for trachelectomy specimens (16.5%); never (63.1%)
How often do you perform frozen section for cervical adenocarcinoma?	Always for hysterectomy specimens (0.6%); sometimes for hysterectomy specimens (28.4%); always for trachelectomy specimens (13.1%); sometimes for trachelectomy specimens (23.3%); never (53.4%)
How many sections of tumor do you examine at frozen section?	0 (52.8%); 1 (23.3%); 2 (14.2%); >2 (9.7%)
Which of the following do you report at the time of frozen section?	Tumor size (17.6%); tumor type (23.3%); tumor grade (11.9%); depth of invasion (23.3%); presence/ absence of lymphovascular invasion (6.8%); other parameters (23.9%); do not perform intraoperative evaluation (51.7%)
Do you evaluate the ovaries and fallopian tubes at intraoperative assessment for cervical adenocarcinoma?	Gross examination only (23.9%); microscopic examination only if gross abnormality (17%); microscopic examination always (1.1%); no (58%)

**TABLE 4:** Questions and responses regarding tumor measurements

How do you report tumor dimensions?	Gross measurement only (5.2%); microscopic measurement only (5.1%); separate gross and microscopic measurements (40.3%); single measurement based on combination of gross and microscopic (49.4%)
What tumor measurements do you report?	Only one single maximum dimension (9.1%); depth of invasion (86.9%); maximum horizontal spread in any direction (44.9%); maximum horizontal spread in 2 directions (44.9%); other (11.9%)
Do you report tumor volume?	Yes (11.4%); no (88.6%)
In reporting the horizontal tumor dimensions, do you include the measurements from prior resections?	Yes—by adding together the maximum horizontal dimension in each specimen (19.3%); yes—by using the single maximum horizontal dimension from either specimen (26.7%); yes—other method (9.1%); no (42%)
In reporting depth of invasion, do you include the measurements from prior resections?	Yes—by adding the maximum depth in each specimen (11.9%); yes—by using the single maximum depth from either specimen (39.2%); yes (other method) (5.9%); no (43%)
For an exophytic tumor that does not invade the underlying stroma or only minimally invades the stroma, how do you report depth of invasion?	Classify as adenocarcinoma in situ (1.2%); report only the depth of stromal invasion, excluding the exophytic component (47.7%); report only the tumor thickness without depth of invasion (19.3%); report the tumor thickness and document this as depth of invasion (13.6%); other (18.2%)
Do you report if the tumor has multiple foci of invasion?	Yes (66.5%); no (33.5%)

**TABLE 5:** Questions and responses regarding tumor grading and typing

Do you report a grade for cervical adenocarcinomas?	Yes—using the grading system for primary endometrial endometrioid carcinoma (48.3%); yes—using a different system (26.7%); no (25%)
Do you report a tumor type for cervical adenocarcinomas?	Yes—using the 2014 WHO Classification (71.6%); yes—using the IECC system (18.8%); yes—HPV-associated versus HPV-independent (5%); yes—using a different system (3%); no (1.6%)
Do you use immunohistochemistry or HPV-testing to determine the tumor type?	Always (22.7%); sometimes (71.6%); never (5.7%)
Do you use these terms as a tumor type for primary cervical adenocarcinoma?	Villoglandular adenocarcinoma (56.8%); serous carcinoma (41.5%); endometrioid carcinoma (52.3%); adenosquamous carcinoma (85.2%)

HPV indicates human papillomavirus.

**TABLE 6:** Questions and responses regarding lymphovascular space invasion and patterns of tumor invasion

Do you report the presence or absence of lymphovascular invasion in cervical adenocarcinomas?	Yes (96.6%); if present it is reported, but if absent this is not commented upon (3.4%); no (0%)
Do you use immunohistochemistry to diagnose lymphovascular invasion?	Yes—even if the findings on H/E are classic (3.9%); yes—but only if the findings on H/E are equivocal (64.8%); no (31.3%)
Do you report the extent of lymphovascular invasion if present?	Yes—(50.6%); no (49.4%)
Do you distinguish between lymphatic and blood vessel invasion if present?	Yes (26.1%); no (73.9%)
Do you report involvement of the uterine corpus by cervical adenocarcinoma?	Yes and I distinguish if it involves the endometrium and/or myometrium (81.8%); yes but I do not distinguish if it involves the endometrium and/ or myometrium (17%); no (1.2%)
Do you routinely report the Silva pattern of invasion in cervical adenocarcinomas?	Yes—for all tumors (12.5%); yes—but only for HPV-associated tumors (33.5%); yes—but only for HPV-independent tumors (1.2%); no (40.9%); I am not familiar with this (11.9%)
Are you clinicians familiar with Silva patterns of invasion?	Yes and I know they incorporate this into decision making (9.1%); yes but I know they do not incorporate this into decision making (19.9%); yes but I do not know if they incorporate this into decision making (18.8%); I do not know (21%); no (31.3%)
Do you report microcystic elongated and fragmented pattern of invasion in cervical adenocarcinomas?	Yes (18.8%); no (47.2%); I have never observed a case but would likely report it if I saw it (25.6%); I have never observed a case but would likely not report it if I saw it (8.5%)

H/E indicates hematoxylin and eosin; HPV, human papillomavirus.

**TABLE 7:** Questions and responses regarding lymph nodes

Do you report the number of lymph nodes in each separately submitted anatomic site (eg, right and left pelvic)?	Yes (96.6%); no (3.4%)
Do you submit all the slices of grossly normal lymph nodes entirely for histologic examination?	Yes, including all extranodal fat (46.6%); yes, not including extranodal fat (47.2%); no (6.2%)
How do submit lymph nodes that contain grossly obvious tumor?	Entirely (27.8%); representative sections (72.2%)
How many nodes are submitted per cassette?	Depends on size (92.6%); 1 (4.6%), 2 (0.8%); 2+ (2%)
Do you report the number of lymph nodes per cassette in the tissue block key?	Yes (88.1%); no (11.9%)
Do you report the number of metastatic lymph nodes in each separately submitted anatomical site?	Yes (98.3%); no (1.7%)
Do you report the maximum size of the metastatic cancer in a lymph node?	Yes (73.3%); no (26.7%)
How do you stage a lymph node that contains isolated tumor cells?	Isolated tumor cells. AJCC stage Pn0 i+ (76.7%); positive for metastatic cancer. AJCC stage pN1 (14.2%); positive for micrometastatic cancer. AJCC stage pN1mic (8%); benign lymph node AJCC pN0 (1.1%)
Do you report on extranodal extension by tumor?	Yes but only if present (52.8%); yes (42.6%); no (4.6%)
For lymph nodes that are negative on hematoxylin and eosin, do you automatically perform cytokeratin staining?	Yes (2.8%): no (97.2%)
Do your clinicians submit sentinel lymph nodes in cases of cervical adenocarcinoma?	Yes (routinely) (21%); yes on occasions (43.2%); no (35.8%)
If sentinel nodes are evaluated, do you perform a frozen section?	Yes always (23.3%); yes if grossly suspicious (9.7%); no (67%)
If sentinel nodes are evaluated, do you use a specific ultrastaging protocol for specimen processing?	Yes (same as for sentinel nodes for endometrium and vulva) (63.1%); yes (different from endometrium and vulva) (3.4%); do not receive sentinel nodes (33.5%)

**TABLE 8:** Questions and responses regarding tumor staging

Which staging system do you use for cervical adenocarcinomas?	TNM (AJCC or UICC) (51.7%); FIGO 2018 (34.1%); FIGO 2009 (4.5%); stage not reported on pathology reports (9.7%)
Do you use the term “microinvasive” when reporting cervical adenocarcinomas?	No (68.8%); yes—or cases that are FIGO IA1/TNM pT1a1 (24.4%); yes—for cases that are FIGO IA1 or 2/TNM pTa1 or 2 (6.8%)
How do you distinguish between when tumor is in the deep cervical wall versus when tumor is in the parametrium?	Tumor extends beyond fibrous stroma of cervical wall (55.8%); need to see tumor extending into adipose tissue (39.8%); other (4.4%)
If tumor extends beyond the cervical stroma at the anterior (paracervical) position (12 o’clock) or the posterior (paracervical) position (6 o’clock), how do you report this?	Parametrial spread (FIGO IIB, pT2b) (39.8%); paracervical spread but does not upstage to stage II (49.4%); other (10.8%)
What stage do you report if there is ovarian metastasis of cervical adenocarcinoma?	Base the stage on all other factors except the ovary and provide a separate comment on the report (73.3%); do not routinely report stage for cervical cancer (15.3%); upstage the tumor (explain how in comments) (11.4%)

**TABLE 9:** Questions and responses regarding ancillary studies

Do you perform p16 staining on all cervical adenocarcinomas?	Yes—in all cases (27.3%); only if needed to resolve uncertain H/E findings regarding primary origin of the tumor or tumor type (63.1%); no (9.7%)
Do you routinely perform other immunohistochemical studies in all cervical adenocarcinomas?	Yes—in all cases (6.2%); only if needed to resolve uncertain H/E findings regarding primary origin of the tumor or tumor type (84.7%); no (9.1%)
Do you use immunohistochemistry to confirm the diagnosis of gastric-type, clear cell or mesonephric carcinoma?	Yes—in all cases (56.8%); only if needed to resolve uncertain H/E findings regarding tumor type (40.3%); no (2.9%)
Do you perform high-risk HPV testing in cervical adenocarcinomas?	Yes—in all cases (4.5%); only if needed to resolve uncertain H/E findings regarding primary origin of the tumor or tumor type (59.7%); I do not have access to high-risk HPV testing for surgical specimens (20.5%); no (15.3%)
Do you perform mismatch repair staining in cervical adenocarcinomas?	Yes (in all cases) (4%); yes (only in HPV-associated tumors) (0.7%); yes (only in HPV-independent tumors) (6.7%); no (88.6%)
Do you order PD-L1 staining in cervical adenocarcinomas?	Yes—performed in my lab and interpreted by me (18.4%); yes—but the test is sent out to a separate commercial or academic lab (17.8%); no (63.8%)
Which scenarios do you order PD-L1 staining in cervical adenocarcinomas?	All primary tumors (2.8%); all metastatic tumors (4%); non–HPV-associated primary tumors (1.1%); HPV-associated primary tumors (0.6%); case by case basis when clinician requests (60.2%); never (35.8%)
How do you report PD-L1 staining?	Percentage of positive tumor cells (13.6%); combined positive score (tumor cells and immune cells) (22.7%); other (2.3%); the test is performed and reported by a separate lab (25.6%); I do not order this test (35.8%)
Do you use biomarkers to resolve uncertainty in cases of primary cervical versus primary endometrial adenocarcinoma?	p16 (96%); CEA (63.6%); vimentin (76.7%); estrogen receptor (91.5%); high-risk HPV testing (51.1%); do not use biomarkers in this scenario (1.1%)

H/E indicates hematoxylin and eosin; HPV, human papillomavirus.

The demographics of the respondents and general questions with responses are shown in Table 1.

Questions and responses regarding gross examination and specimen processing are shown in Table 2.

Questions and responses regarding intraoperative tumor assessment are shown in Table 3.

Questions and responses regarding tumor measurement are shown in Table 4.

Questions and responses regarding tumor grading and typing are shown in Table 5.

Questions and responses regarding lymphovascular space invasion (LVSI) and “patterns” of tumor invasion are shown in Table 6.

Questions and responses regarding lymph node reporting are shown in Table 7.

Questions and responses regarding tumor staging are shown in Table 8.

Questions and responses regarding ancillary studies are shown in Table 9.

## DISCUSSION

The results of this extensive survey (undertaken by 38.5% of the ISGyP membership) were useful in collecting baseline information on current practice regarding pathologic reporting of cervical adenocarcinomas. As expected, the results showed significant variation in many areas and some of them are discussed in detail in other reviews in this issue of the journal where recommendations are provided. In all, 77.3% of respondents to the survey worked in academic institutions and 86.9% only practice gynecologic pathology or are oligospecialists with gynecologic pathology an important component of their daily signout.

Participants to the survey were asked whether they were aware of and use the International Collaboration on Cancer Reporting (ICCR) recommendations for cervical cancer The ICCR was instituted in 2011 with the goal of reducing global burden of cancer dataset development and reduplication of efforts by different international institutions that commission, publish and maintain standardized cancer reporting datasets. Many countries expend a great deal of time, effort and resources to produce their own standardized cancer-reporting datasets while other countries lack sufficient pathologist manpower and resources to develop or implement standardized cancer-reporting protocols and benefit from the availability of internationally accredited datasets. The ICCR includes The Royal Colleges of Pathology of Australasia and United Kingdom, the College of American Pathologists (CAP), the Canadian Partnership Against Cancer and the European Society of Pathology (ESP). The ICCR has also formed strategic partnerships with the International Agency for Research on Cancer (IARC) [the organization that is responsible for producing the World Health Organization (WHO) “Blue books” on tumor classification], the organizations responsible for tumor staging [the Union for International Cancer Control (UICC), American Joint Committee on Cancer (AJCC), the International Federation of Obstetricians and Gynaecologists (FIGO)], the ISGyP and the European Organisation for Research and Treatment of Cancer (EORTC). The ICCR has successfully developed most of the gynecologic cancer datasets, including a cervical cancer dataset, in conjunction with ISGyP 8. All of the datasets are evidence-based and were produced by a panel of internationally recognized expert pathologists and a single clinician. The datasets have been subject to international open consultation, and are freely available for worldwide use at the following website: http://www.iccr-cancer.org/datasets. While 80.7% of respondents to the survey were aware of the ICCR recommendations for reporting of cervical cancer, only about half (51.7%) used them. This is likely to reflect the fact that many countries have their own cancer reporting datasets; since a major aim of the ICCR is that the datasets of various countries will follow the ICCR recommendations, many pathologists will be using datasets which are largely based on the ICCR recommendations with some minor local variations. In all, 70.5% of respondents reported using the CAP protocol for reporting cervical cancers.

As expected, the survey revealed significant variation between respondents with regard to grossing and processing of resection specimens. Specimens were predominantly grossed by pathology assistants/laboratory technicians and trainees and to a lesser extent by the pathologists responding to the survey. This is important to know as specimen handling may vary. A large majority of respondents (90.3%) fixed hysterectomy/trachelectomy specimens before grossing. A large majority also inked parametrial and vaginal resection margins, the upper endocervical margin in trachelectomy specimens and intact cervical loop/ cone specimens; inking of fragmented unoriented cervical loop excisions was more variable. A large majority also stated whether cone/loop specimens were in the form of intact loops or strips and provided the measurements of each piece of tissue on the pathology report. Respondents also generally recorded the length of the vaginal cuff, either as a maximum and minimum length or as a single measurement. Most reported a measurement of the parametrial tissues, either as both horizontal/ lateral and vertical extent (most common) or as a single measurement. The site of the tumor within the cervix was usually recorded and most respondents (88.6%) submitted the entire parametrial tissue for histologic examination. The degree of sampling of a macroscopically visible tumor varied but almost all respondents (97.7%) submitted the whole cervix if the tumor was not grossly visible. Most respondents undertook selective sampling of grossly normal lower uterine segment, uterine corpus, fallopian tubes and ovaries, although 45.5% submitted the entire fallopian tubes, mostly using a SEE-FIM protocol. Only a small number of respondents (10.8%) used large tissue blocks (macroblocks).

A minority of respondents routinely performed intraoperative assessment (gross examination and/or frozen section) of hysterectomy or trachelectomy specimens for cervical adenocarcinoma. When performing frozen section, most examined only a single tumor section. The most common parameters assessed at frozen section were tumor type (23.3%), depth of invasion (23.3%) and tumor size (17.6%). Most respondents did not assess the adnexa at intraoperative assessment and when they did this usually comprised gross examination only with microscopic examination reserved for cases where there was a macroscopic abnormality.

One of the most problematic areas in reporting of cervical carcinomas is the provision of accurate tumor dimensions both in microscopic and in large neoplasms. There are several scenarios where accurate tumor measurement is crucial for staging, management or prognostication 8. There are multiple areas of difficulty in measuring cervical carcinomas and in glandular tumors, there may be specific difficulties in distinguishing between in situ and invasive adenocarcinoma. Some tumors, including small carcinomas, can only be measured microscopically while larger tumors may be best measured grossly. Several reporting datasets include both gross and microscopic measurements and this can be confusing for clinicians if they differ. The ICCR recommends that only a single set of tumor measurements be provided and these should be based on a combination of gross and microscopic examination 8. About half of respondents (49.4%) employed this practice and a significant number (40.3%) provided separate gross and microscopic measurements. Most respondents (86.9%) reported a depth of invasion on their reports. 44.9% reported 2 horizontal measurements and 44.9% reported only a single maximum horizontal dimension. Only 11.4% of respondents reported tumor volume. Another controversial area in measuring cervical carcinomas is whether to take into account the measurements in prior resections, for example, when a trachelectomy or hysterectomy is performed following a loop excision. The ICCR recommends adding together the maximum horizontal dimension in both specimens recognizing that this will almost certainly overestimate the maximum horizontal dimension; it recommends that the maximum depth of invasion in either specimen be taken as the final depth of invasion (and that the 2 depths should not be added together) 8. This proved a very controversial area in the survey with marked variation between respondents. Only 19.3% used the ICCR recommendations for determining the maximum horizontal dimension and 39.2% for determining the depth of invasion. Another controversial area was the approach taken to measure an exophytic tumor that does not invade or only minimally invades the underlying stroma. There was marked variability among the respondents; 47.7% reported the depth of stromal invasion excluding the exophytic component, 19.3% reported only the tumor thickness without measuring the depth of invasion, and 13.6% reported the tumor thickness and equated this to the depth of invasion. Measurement of multifocal carcinomas, which anecdotally is more common in squamous carcinomas than adenocarcinomas, is also a problematic area. Most respondents to the survey (66.5%) reported multifocal carcinomas while 33.5% did not. Recommendations have been proposed for reporting multifocal squamous carcinomas 8–10 and these have been provisionally endorsed by ICCR but similar recommendations do not exist for cervical adenocarcinomas.

It is widely acknowledged that the current 2014 WHO Classification of cervical adenocarcinomas is suboptimal and includes poorly reproducible categories, with diagnosis largely based on subjective morphological features 11. Several of these categories, such as villoglandular, serous and endometrioid carcinoma, either in all likelihood do not exist or represent morphologic variants of HPV-associated adenocarcinoma. This has resulted in a new International Endocervical adenocarcinoma Criteria and Classification (IECC) proposal for the classification of cervical adenocarcinomas, largely incorporated into the upcoming 2020 WHO Classification, and predominantly based on the correlation of morphologic features with oncogenic high-risk HPV 12–15. While most respondents (71.6%) reported using the 2014 WHO Classification, others use the IECC system or even other systems. Regarding reporting those controversial categories above, 56.8%, 41.5%, and 52.3% use the categories of villoglandular adenocarcinoma, serous carcinoma, and endometrioid carcinoma, respectively. There is no validated grading system for cervical adenocarcinomas; 48.3% of respondents reported using the grading system for endometrioid carcinomas of the uterine corpus, as suggested by the ICCR 8, while 25% did not grade these neoplasms. Given the different morphologic types of cervical adenocarcinoma (HPV-associated and various HPV-independent), it is doubtful whether a single grading system could be applicable to cervical adenocarcinomas as a whole.

Almost all respondents report LVSI and almost all do not routinely use immunohistochemistry to help identify it unless the features are equivocal on examination of hematoxylin and eosin–stained slides. Most respondents (73.9%) did not distinguish between lymphatic and blood vessel invasion. The extent of LVSI is now widely reported in endometrial carcinomas since it has been demonstrated to be a very important prognostic parameter 16,17. In a large series of uterine endometrioid carcinomas from the PORTEC trials, substantial LVSI, in contrast to focal or absent LVSI, was the strongest independent prognostic factor for pelvic regional recurrence, distant metastases, and overall survival 16. However, the extent of LVSI has not been proven to be of prognostic significance in cervical carcinomas. In spite of a lack of standard recommendations in reporting LVSI, just over half of the respondents (50.6%) provided some comment regarding its extent on the pathology report. Review of the comments revealed a wide range of methods of recording LVSI ranging from documenting the exact number of vessels involved to semiquantitative assessment (focal, multifocal, extensive, etc.). Almost all respondents (98.8%) reported uterine corpus involvement by cervical adenocarcinoma even though this does not result in upstaging of the tumor and a significant majority of respondents (81.8%) specifically distinguished between endometrial and myometrial involvement.

The Silva pattern-based classification of invasion in HPV-associated adenocarcinomas (discussed in another review in this issue) has received a lot of attention in the literature in recent years 18. Although approximately half (47.2%) of respondents recorded the Silva pattern of invasion on their pathology reports (mostly for HPV-associated adenocarcinomas only but sometimes for all adenocarcinomas), only 9.1% stated that their clinicians used this information in their decision-making. This is likely to reflect the fact that this system is largely unknown to clinicians (outside of a few major centers), although it seems that Silva pattern C seems to have an impact predicting lymph node metastasis while pattern A seems to correlate with lack of lymph node metastasis independently of depth of stromal invasion. Suggestions on how the Silva patterns of invasion can be integrated into the clinical management of patients with cervical adenocarcinoma is discussed in a later review. A lesser number of respondents (18.8%) reported microcystic elongated and fragmented pattern of cervical stromal involvement. This pattern of invasion is much more characteristic of endometrioid carcinomas of the corpus exhibiting myometrial invasion but has also been reported in cervical adenocarcinomas 19.

Lymph node involvement is one of the most important prognostic factors in cervical carcinoma. Almost all respondents reported the number of lymph nodes retrieved from each anatomical site and the number from each anatomical site involved by tumor. Almost all respondents (93.8%) submitted grossly normal nodes in their entirety for histologic examination and approximately half included the extranodal fat; most (72.2%) submitted representative sections of grossly involved nodes. The number of nodes submitted per cassette depended on the size of the nodes and most reported the number of nodes per cassette in the tissue block key. Most respondents (73.3%) reported the maximum size of a metastatic deposit within a node and almost all reported extranodal extension if present. Only a small minority of respondents (2.8%) automatically performed cytokeratin staining on nodes that were negative on hematoxylin and eosin staining. The staging of a lymph node containing isolated tumor cells was controversial. Answers proffered were AJCC stage pN0i+ (76.7%), AJCC stage pN1 (14.2%), AJCC stage pN1mic (8%), and benign lymph node AJCC pN0 (1.1%). Respondents reported variably that their clinicians submitted sentinel lymph nodes but most did not do so routinely. Only a minority of respondents performed frozen sections on sentinel lymph nodes and most used a specific ultrastaging protocol, which was usually the same as used for endometrial and vulval sentinel lymph nodes.

Regarding staging systems used by respondents for cervical carcinomas, most (51.7%) utilized TNM (AJCC or UICC) and this is likely to reflect the fact that TNM staging is mandated in tumor reporting in some countries, such as the United States. The FIGO staging system is widely used in other countries 20 and some respondents (34.1%) used FIGO 2018 and a smaller number (4.5%) use FIGO 2009. Others (9.7%) stated that they do not include a tumor stage on their pathology reports. Broadly, TNM and FIGO staging for gynecologic carcinomas are similar with TNM adopting the updated FIGO staging systems sometime after they are introduced. Currently, the systems differ for cervical carcinoma in that TNM has not been updated to reflect the new 2018 FIGO staging system 7. The term “microinvasive carcinoma” does not appear in the FIGO or TNM staging systems for cervical cancer. Furthermore, use of the term “microinvasive carcinoma” has different connotations in different geographical areas. Thus, in order to avoid confusion, it is recommended by the ICCR and other authorities to avoid using the term “microinvasive carcinoma” for cervical carcinomas but o use the specific stage 8. Most respondents to the survey (68.8%) stated that they did not use the term “microinvasive carcinoma.” Most who did use this term applied it for stage IA1 carcinomas only but a smaller percentage used for all stage IA carcinomas.

Ovarian involvement by cervical carcinoma is uncommon but is more common with adenocarcinomas than squamous cell carcinomas. For example, in a large study of 1965 patients by Landoni et al. 21, ovarian metastases were present in 16 (0.9%) and were more common in adenocarcinoma (2.4%) than squamous carcinoma (0.5%). In addition, HPV-independent cervical adenocarcinomas, especially gastric-type, are particularly prone to involve the ovary 22–24. Staging of a cervical carcinoma with ovarian involvement is controversial. Anecdotally some pathologists and clinicians upstage tumors with ovarian involvement to stage II, III or IV. However, ovarian involvement was not mentioned in the 2009 FIGO staging system and the updated FIGO 2018 staging system states “presently ovarian involvement does not change the stage” 7. Thus, a cervical carcinoma involving the ovary but otherwise confined to the cervix, should be FIGO stage I. Furthermore, FIGO 2018 states that “ovarian involvement does not change stage due to low incidence in early stage disease (<1% in squamous cell carcinoma, <5% in other types), common association with other high-risk features, and limited data on impact on survival as an independent risk factor.” However, this remains a controversial issue and most clinicians would offer adjuvant therapy to patients with ovarian involvement, even if the other parameters did not warrant this action. In our survey, 73.3% of respondents indicated that they would base stage on all other parameters except ovarian involvement and provide a separate comment on the report while 11.4% indicated they would upstage the tumor; the stage suggested by the latter group ranged from II to IV. Another controversial area is how to stage a cervical tumor extending beyond the cervical stroma at the anterior (paracervical) position (12 o’clock) or the posterior (paracervical) position (6 o’clock). 39.8% of respondents reported this finding as parametrial spread (FIGO IIB, pT2b) and 49.4% as paracervical spread which does not upstage the tumor to stage II.

Respondents stated that they commonly used ancillary immunohistochemical studies (especially p16) in typing cervical adenocarcinomas, especially if the findings were uncertain on hematoxylin and eosin–stained sections. HPV studies were generally undertaken only in selected cases to help confirm the tumor type or determine the site of origin. Immunohistochemistry was commonly used to assist in confirming a diagnosis of an HPV-independent cervical adenocarcinoma (gastric-type, clear cell, mesonephric). Respondents also commonly used biomarkers to resolve uncertainty in primary cervical versus endometrial origin. In this scenario, the most commonly used biomarkers were p16 (96%), CEA (63.6%), vimentin (76.7%), estrogen receptor (91.5%), and high-risk HPV testing (55.1%). Mismatch repair immunohistochemistry was generally not performed by pathologists on cervical adenocarcinomas (88.6%); those who performed mismatch repair immunohistochemistry indicated that this was most commonly for HPV-independent adenocarcinomas. PD-L1 immunohistochemistry was variably undertaken and most commonly at the request of the clinician in an individual case. The method of reporting PD-L1 staining was also variable.

As with all surveys of this nature, there is the possibility of a degree of “self-selection” in those responding to the survey in that so-called experts may preferentially undertake the survey; however, the large number and wide geographical variation of the respondents suggests that the responses are representative of gynecologic pathologists in general.

In summary, as expected the results of our extensive survey revealed areas of uniformity but also areas of substantial variation in the grossing, processing and reporting of cervical adenocarcinomas. The responses to the survey assisted in developing the subsequent recommendations presented in other reviews in this issue.
